# Gene expression profile and bioinformatics analysis revealed key molecular characteristics of chordoma—before and after TNF- a treatment

**DOI:** 10.1097/MD.0000000000018790

**Published:** 2020-01-17

**Authors:** Guoyong Xu, Chong Liu, Tuo Liang, Zide Zhang, Jie Jiang, Jiarui Chen, Jiang Xue, Haopeng Zeng, Zhaojun Lu, Xinli Zhan

**Affiliations:** a Guangxi Medical University; b Spine and Osteopathy Ward, The First Affiliated Hospital of Guangxi Medical University, Nanning, PR China.

**Keywords:** bioinformatics, chordoma, differentially expressed genes, FGF2, KDR, PDGFRB, Pi3k-Akt signaling pathway

## Abstract

**Background:**

Chordoma is a rare malignant tumor with limited treatment. Recent studies have shown that the proliferation and invasion ability of chordoma after Tumor necrosis factor alpha (TNF-α) treatment is enhanced, which may activate the gene pathway involved in the development of chordoma. This study tends to identify differentially expressed genes (DEGs) before and after treatment of TNF-α in chordoma cell line, providing a new target for future molecular therapy of chordoma.

**Methods:**

The gene expression profile of GSE101867 was downloaded from the Gene Expression Omnibus database, and the differentially expressed genes were obtained using GEO2R. Based on the CLUEGO plugin in Cytoscape, DEGs functionality and enrichment analysis. A protein-protein interaction (PPI) network was constructed using Cytoscape based on data collected from the STRING online dataset. The Hub genes are selected from the CytoHubba, the first 20 genes that coexist with the KEGG tumor-related pathway.

**Results:**

A total of 560 genes, including 304 up-regulated genes and 256 down-regulated genes, were selected as DEGs. Obviously, GO analysis shows that up-regulated and down-regulated DEGs are mainly enriched in biological processes such as synaptic tissue, cell adhesion, extracellular matrix organization and skeletal system development. DEGs are mainly enriched in tumor-associated pathways such as Pi3k-akt Signal path, Rap1 signal path. Three key genes were identified: PDGFRB, KDR, FGF2. All of these genes are involved in the tumor-associated pathways described previously.

**Conclusion:**

This study is helpful in understanding the molecular characteristics of chordoma development. Hub genes PDGFRB, KDR, FGF2 and pi3k-akt signaling pathway, Rap1 signaling pathway will become a new target for the future treatment of chordoma.

## 1 Introduction

Chordoma is a malignant bone tumor with a high recurrence rate, most occur in the skull base and the sacrum.^[1,2]^ It has limited knowledge of molecular pathogenesis and lacks effective chemotherapy drugs. The current treatment methods are mainly active surgical resection followed by radiotherapy.^[3]^ Although newer auxiliary radiotherapy methods have been shown to reduce the recurrence rate,^[4]^ the persistence of radioresistant chordoma cells has led to a high recurrence associated with chordoma, especially in the sacrum. During past few years, medical researchers have been absorbing on the molecular structural characteristics of chordoma to identify drug targets,^[5]^ help to treat and reduce recurrence rates, and improve patients’ survival rate and quality of life.

Clinically, surgery is the main treatment option. As surgical resection is limited by neurovascular damage, the recurrence rate of chordoma is more than 40%.^[6]^ Also, chordoma has a strong resistance to chemotherapy and radiotherapy, leading to a decline in patients’ quality of life and poor prognosis.^[7]^ The overall median survival time of chordoma is estimated to be approximately 6 years, with a 5-year survival rate of 70% and a 10-year survival rate of 40%.^[8]^

Recently, a study has shown that the proliferation and invasiveness of the sacral chordoma cell line after TNF-α treatment are enhanced, and a series of experiments have proven that TNF-α can be used as a prognostic indicator of chordoma develpment and inflammatory chordoma promotion.^[9]^ This may be related to the genetic pathways involved in the development of chordoma activated by TNF-α. Considering the treatment of TNF-α as an inducing factor, the differential genes before and after TNF-α treatment can be equated with the key genes for the development of chordoma.

In this study, we applied a variety of bioinformatics analysis methods on the expression profile data provided by Omer Faruk Bayrak et al.^[9]^ Our goal is to reveal molecules that are abnormally expressed in the development of chordoma to help understanding the biological characteristics of chordoma development and to provide new molecular targets for the treatment of chordoma in the future.

## 2 Methods and materials

### 2.1 Microarray data

The GSE101867 gene expression data set is derived from GPL17692 (Affymetrix Human Gene 2.1 ST Array (http://www.ncbi.nlm.nih.gov/geo/) which contains 10 samples of two sacral chordoma cell line groups, of which three U-CH1 untreated specimens, three MUG-Chor1 untreated specimens, two U-CH1 specimens after one year of TNF-α treatment, and two MUG-Chor1 specimen after TNF-α treatment for one year. These chordoma cell lines originate from the sacrum. U-CH1 related specimens were arranged into two groups: no treatment control group (3 cases) and after one year of TNF-α treatment group (2 cases). This study was based on data from an open database. Ethics and patient consent are not applicable.

### 2.2 Data processing

DEGs were filtered using GEO2R (http://www. Ncbi.nlm.nih.gov/geo/geo2r/), an online analysis tool for identifying differentially expressed genes.^[10]^ The results of the analysis are saved in a file format, the cut-off criteria of DEGs is set as: adj*P* < .05, |log fold-change|>2.

### 2.3 Gene ID conversion

The differential gene original codes (GB_ACC) filtered and analyzed by GEO2R were converted into an official gene symbol using the DAVID database^[11]^ (DAVID version: 6.8).

### 2.4 GO and KEGG enrichment analysis

DEGs’ GO and KEGG enrichment analysis were performed using the plugin ClueGO^[12]^ (version 2.5.4) in the Cytoscape software^[13]^ (3.7.1) The standard setting with statistically significant difference was *P* < .05 and k value was 1.0.

### 2.5 PPI network construction

Integrate the PPI network. The interaction between DEGs was evaluated using the string database^[14]^ (version 11.0); a composite score > 0.4 was considered a statistically significant interaction. Subsequently, the analysis results of PPI network were loaded into Cytoscape software for visual adjustment.

### 2.6 Identification of hub genes

CytoHubba^[15]^ (version 0.1) is a plugin for identifying key genes from PPI network in Cytoscape. Considering the CytoHubba calculation ordering will count the genes for non-tumor related pathways that would confuse our purpose and results. We took the top 20 genes after Degree calculation method and the tumor-associated pathway-related genes to take Venn intersection, and the genes coexisting with the kegg tumor-related pathway were considered as hub genes.

## 3 Results

### 3.1 Identify and assess differential expression levels of DEGs

**Figure 1:**
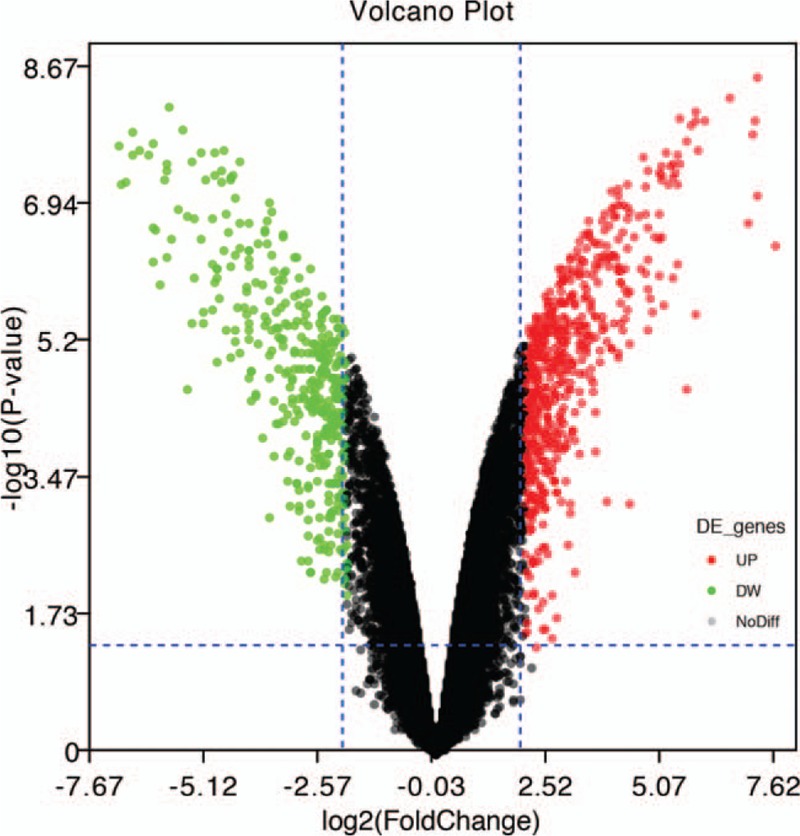
Volcano map of all DEGs, screening criteria: *P* < .05 and |logFC |> 2.

**Figure 2:**
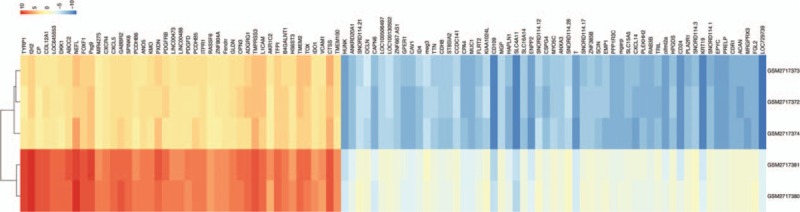
Heat map of the top 100 differentially expressed genes. Red indicates upregulated genes and blue indicates downregulated genes.

According to the analysis of GEO2R and the results of the DAVID database conversion gene ID, a total of 560 differentially expressed genes were identified between untreated and one year of TNF-α treatment in U-CH1 cell line, including 304 up-regulated genes and 256 down-regulated genes. Furthermore, the volcano map and the heat map show that DEGs have a significant effect in the difference between the two groups (Figs. 1 and 2).

### 3.2 GO functional enrichment analysis

**Figure 3:**
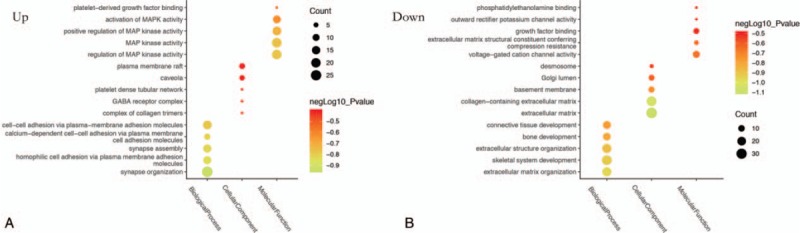
A and B are Top 5 GO functional enrichment analysis results from up and down regulate DEGs.

GO functional enrichment analysis of differentially expressed genes by “ClueGO”. The results indicate that both up and down regulated DEGs are mainly enriched in biological processes. In Figure 3, A and B are the bubble maps of top five items of GO functional enrichment analysis of up-regulated and down-regulated genes in “ClueGO”, respectively (standard cut-off *P* < .05). As is evident from Figure 3, the related cellular components of up-regulated DEGs are mainly enriched in collagen trimer complex, platelet-dense tubular network. In biological process analysis, the up-regulated DEGs are mainly enriched in synaptic tissue and cell adhesion processes. Besides, in molecular function (MF) analysis, up-regulated DEGs are mainly enriched in the regulation of MAP kinase activity and platelet-derived growth factor binding. However, for down-regulated DEGs, they are enriched in the extracellular matrix, the basement membrane, and the Golgi cavity(CC). In biological process analysis, they are enriched in extracellular matrix tissues, skeletal system development. About molecular function, they are mainly involved in voltage-gated cation channel activity, growth factor binding, and phosphatidylethanolamine binding.

### 3.3 KEGG pathway enrichment analysis

**Figure 4:**
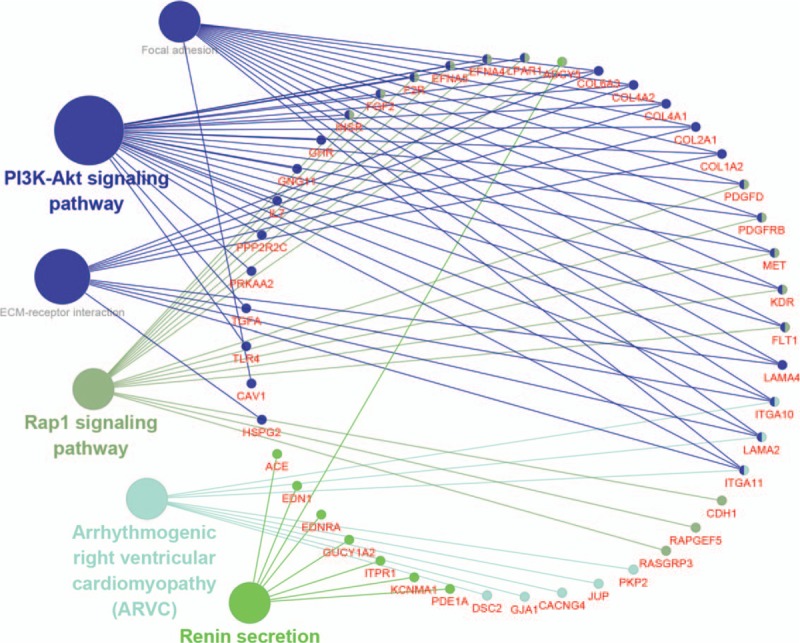
KEGG enrichment analysis results of all DEGs.(*P* < .05).

To more intuitively show the enrichment analysis of DEGs in the KEGG pathways, we concentrated all DEGs together for analysis(*P* < .05) (Fig. 4). It can be clearly seen from the color classification in the figure that DEGs are mainly enriched in four type pathways, and the key pathways related to tumors are Pi3k-Akt signaling pathway and Rap1 signaling pathway.

### 3.4 Identification of hub genes

**Figure 5:**
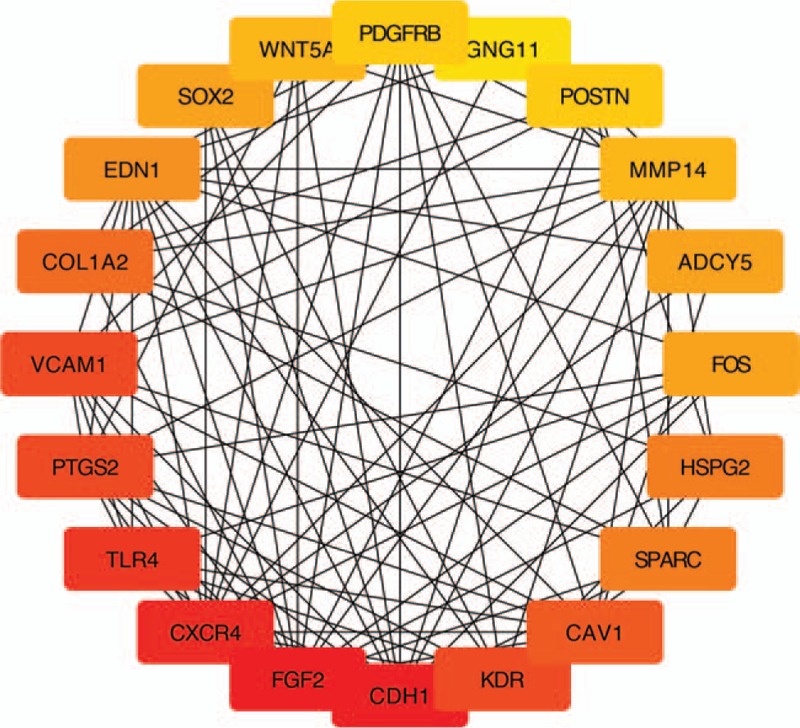
The top 20 genes in Degree score from cytoHubba.

**Figure 6:**
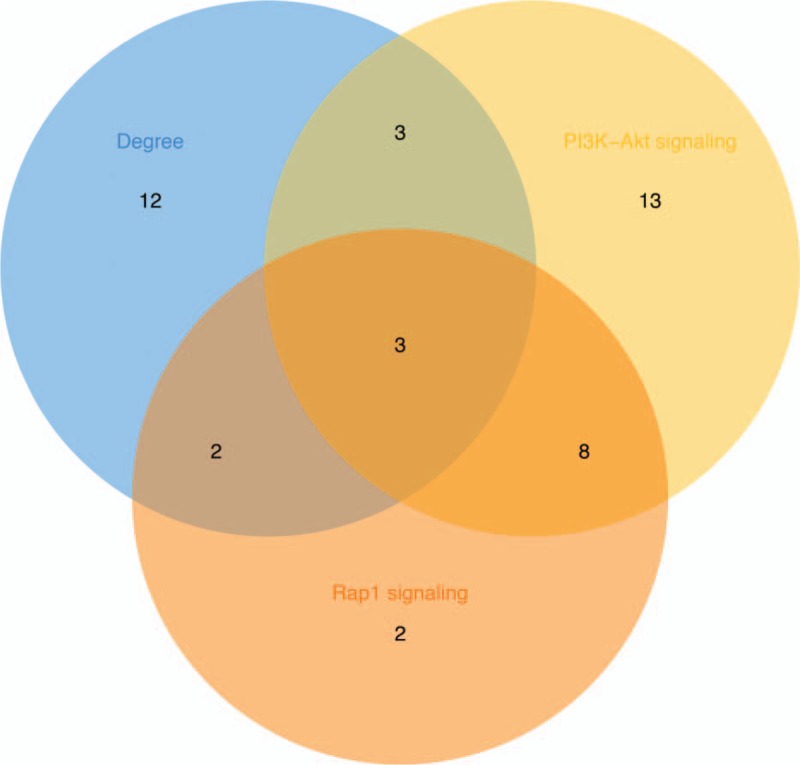
Venn intersection map of tumor-related pathway associated genes and top 20 genes in Degree score.

The top 20 genes were gotten by the CytoHubba (Fig. 5). Next, we derived the data from the kegg analysis in Cluego and used the relevant genes in the tumor-associated pathway (Table 1) for the Venn crossover (Fig. 6). As a result, three hub genes were identified (Table 2).

**Table 1:**
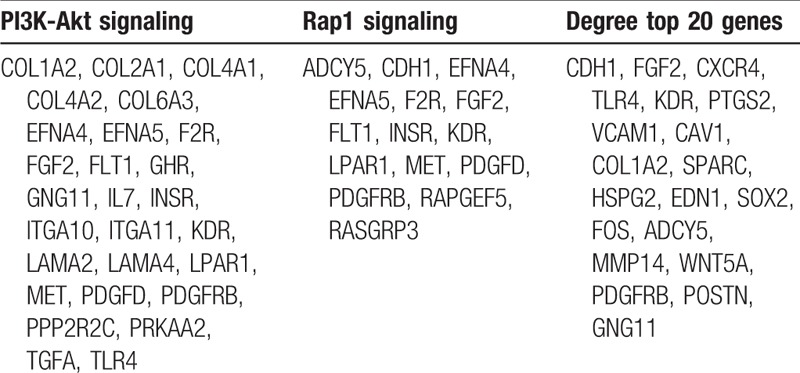
Associated genes in the tumor pathway and Degree top 20 gene.

**Table 2:**
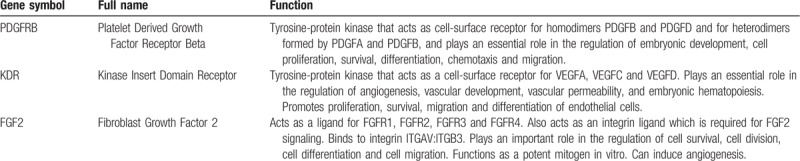
Function of 3 hub genes.

### 3.5 PPI network construction

**Figure 7:**
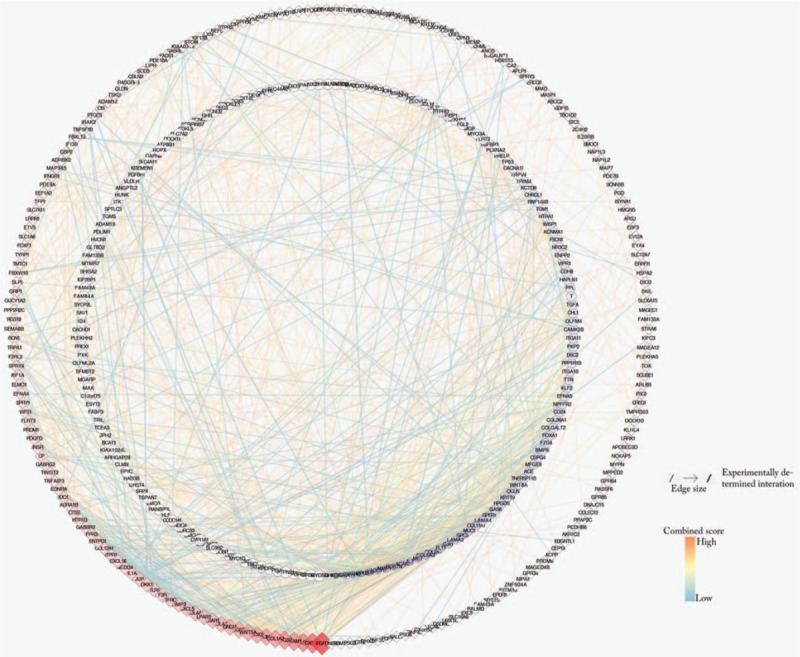
All DEGs PPI networks are visualized in Cytoscape. The red diamonds represent the up-regulated DEGs and the blue balls represent the down-regulated DEGs. As the degree score increases, the color of the ball gradually changes from light blue to dark blue, and their size gradually becomes smaller. The color of the diamond gradually changes from light red to deep red, and their size gradually increases.

The STRING database is used for all DEGs PPI network construction. The data is then entered into Cytoscape to adjust the visualization, which has 397 nodes and 1234 edges. (Fig. 7).

## 4 Discussion

Chordoma is a malignant bone tumor with recurrence and potential metastasis tendency. The patient has a poor prognosis, accounting for 4% of all primary bone tumors and 20% of primary spinal tumors.^[12]^ About 50% to 60% of chordoma occur in the sacrum. Since human notochord degrades in late fetal development and early postpartum, there are no clear adult tissue counterparts in comparative studies.^[16]^ As a result, the biological characteristics of chordoma have not been fully understood so far. Finding key genes and pathways in the development of chordoma can greatly facilitate diagnosis, help with treatment and prognosis assessment.

Molecular targeted therapy is a new direction for cancer therapy. Compared with traditional chemotherapy, targeted therapeutic drugs inhibit specific molecules or signal pathways to inhibit tumor growth and promote tumor cell apoptosis. According to the available evidence, Tyrosine kinases are class of cellular signaling molecules that regulate cell proliferation, differentiation, growth and apoptosis. Chordoma has different degrees of expression in receptor tyrosine kinases (RTK). Clinical reports of RTK inhibitor chemotherapy in patients with chordoma can cause tumors to stagnate and even shrink tumors.^[17]^ The epidermal growth factor receptor (EGFR) is also one of the RTKs. EGFR is expressed in the cell membrane, and the expression rate of chordoma is 35% to 100%. EGFR expression was significantly higher in recurrent chordomas than in primary chordomas.^[18]^ Chordoma cell lines and animal models have demonstrated that the use of EGFR inhibitors can effectively inhibit tumor proliferation.^[19]^ Besides, the brachyury gene is a highly conserved gene belonging to the T-box transcription factor family, and a large clinical trial analyzed the expression of brachyury protein in chordoma.^[20]^ Genetic analysis also found that the brachyury gene polymorphism in familial and sporadic chordoma may be associated with the incidence of chordoma, which is common in chordoma.^[21]^ Some studies have attempted to use the method of cellular immunity to breed CD8+ immune cells specific for brachyury protein for immunotherapy of tumors. The clinical efficacy remains to be seen.^[22,23]^

In our study, we identified 560 differential genes, including 304 up-regulated DEGs and 256 down-regulated DEGs. GO analysis showed that both up and down regulated DEGs are mainly enriched in biological processes like synaptic tissue, cell adhesion, extracellular matrix organization and skeletal system development. KEGG enrichment analysis shows that DEGs are mainly enriched in four types of pathways. Among them, such as the PI3K-Akt signaling pathway, Rap1 signaling pathway, Arrhythmogenic right ventricular cardiomyopathy signaling pathway, Renin secretion signaling pathway. The tumor-associated pathway is only PI3K-Akt signaling pathway,^[24]^ Rap1 signaling pathway.^[25]^ Considering the cytoHubba computational ranking will count the genes of the other two non-tumor-related pathways, which will confuse our research. Therefore, we took the top 20 genes in Degree and the tumor-associated pathway-related gene to take Venn intersection, and identified three Hub genes: PDGFRB, KDR and FGF2.

Interestingly, the GeneCards database indicates that PDGFRB, KDR and FGF2 play important roles in cell proliferation, cell survival, cell differentiation, and cell migration (Table 2). The platelet-derived growth factor receptor (PDGFRB), whose encoded protein (cell surface tyrosine kinase receptor) belongs to the platelet-derived growth factor family. The main function is responsible for regulating embryonic development, cell proliferation, survival, differentiation, chemotaxis and migration.^[26,27]^ As a protein-coding gene, the Kinase Insert Domain Receptor (KDR) mainly regulates vascular development, angiogenesis, vascular permeability and embryonic hematopoiesis, and also promotes the survival, proliferation, differentiation and migration of endothelial cells.^[28,29]^ FGF2(fibroblast growth factor 2) belongs to the fibroblast growth factor (FGF) family. As a ligand of FGFR1, FGFR2, FGFR3, and FGFR4, it is mainly responsible for regulating cell division, cell differentiation, and cell migration.^[30,31]^ Besides, experimental analysis has proved that PDGFRB had a different degrees expressed in chordoma.^[32]^ Yixuan Zhai had reported that PDGFRB could regulate the invasion of clival chordoma cells through the mTOR pathway.^[33]^ Recently, Nadia Hindi's team performed a clinical drug trial of imatinib in patients with PDGFB/PDGFRB-positive advanced chordoma and reviewed the demographics, treatment duration, toxicity and response rate by Response Evaluation Criteria in Solid Tumors (RECIST). The results confirmed the anti-tumor activity of imatinib in chordoma.^[34]^ However, although KDR and FGF2 have not been reported to be associated with chordoma progression, they have been shown to be associated with tumor proliferation and invasion in a variety of tumors. Back in the 1990s, overexpression of KDR is associated with colon cancer^[35]^ and intestinal-type gastric cancer.^[36]^ Also, overexpression of FGF2 was found to be associated with the progression of gastric cancer.^[37]^ Strikingly, Ryohei Otani evaluated gene copy number and expression in 27 chordoma specimens by polymerase chain reaction (PCR) confirmed that the activation of PI3K/Akt pathway and the increase of Brachyury copy number are closely related to the overexpression of Brachyury, which seems to be a key event in the growth regulation of chordoma.^[38]^ Moreover, Joseph Schwab confirmed the constitutive activation of PI3K/AKT and mTOR signaling pathways in chordoma by PI3K/mTOR pathway activation and inhibition experiments in chordoma. PI-103 reduces UCH-1 proliferation and induces apoptosis by inhibiting the PI3K / mTOR pathway.^[39]^ Interestingly, although the Rap1 signaling pathway has not been shown to be associated with chordoma, Sitong Zhou recently demonstrated that SHARPIN promotes melanoma migration and invasion by regulating Rap1 and its downstream pathways (including p38 and JNK/c-jun) by activating the Rap1 signaling pathway.^[40]^

## 5 Conclusion

Regarding the treatment of chordoma, the need for effective adjuvant chemotherapy is undoubted.^[41]^ Despite the advancement of surgical methods and advances in surgical techniques, the chordoma after surgical excision still appears local and distant recurrence.^[42]^ Unfortunately, although the advent of carbon ion and proton beam radiotherapy in recent years has been able to control chordomas locally, it does not seem to reduce the recurrence rate of chordomas.^[43,44]^ Our research will help to further understand the molecular characteristics of chordoma development. The Hub genes PDGFRB, KDR, and FGF2 are involved in the pathogenesis of chordoma via the Pi3k-Akt signaling pathway and the Rap1 signaling pathway. This discovery will facilitate future molecularly targeted therapies for chordoma and provide good local control and survival.

## Acknowledgments

We are grateful to Dr. Xinli Zhan (Spine and Osteopathy Ward, The First Affiliated Hospital of Guangxi Medical University,) for his kindly assistance in all stages of the present study.

## Author contributions

**Conceptualization:** Guoyong Xu.

**Data curation:** Guoyong Xu, Tuo Liang, Haopeng Zeng.

**Formal analysis:** Guoyong Xu, Chong Liu.

**Funding acquisition:** Guoyong Xu, Xinli Zhan.

**Investigation:** Guoyong Xu, Chong Liu, Tuo Liang.

**Methodology:** Guoyong Xu, Chong Liu, Jie Jiang.

**Project administration:** Guoyong Xu, Chong Liu.

**Resources:** Guoyong Xu, Zide Zhang.

**Software:** Guoyong Xu, Tuo Liang, Jie Jiang, Xinli Zhan.

**Supervision:** Guoyong Xu, Xinli Zhan.

**Validation:** Guoyong Xu, Chong Liu, Zide Zhang, Jiarui Chen.

**Visualization:** Guoyong Xu, Tuo Liang, Jiang Xue, Zhaojun Lu.

**Writing – original draft:** Guoyong Xu.

**Writing – review & editing:** Guoyong Xu, Chong Liu.

Guoyong Xu orcid: 0000-0003-0636-6831.
